# Addressing Malnutrition in the Aging Population

**DOI:** 10.3390/nu18010070

**Published:** 2025-12-25

**Authors:** Patrizia D’Amelio

**Affiliations:** Service of Geriatric Medicine & Geriatric Rehabilitation, Department of Medicine, University of Lausanne Hospital (CHUV), 1011 Lausanne, Switzerland; patrizia.damelio@chuv.ch

Malnutrition in older adults is a critical yet often underestimated healthcare concern with profound implications for healthy aging. Physiological changes such as reduced appetite, dental problems, and chronic diseases, combined with psychological factors like depression and social isolation, significantly increase the risk of malnutrition. These challenges are compounded by functional limitations and socioeconomic barriers, making malnutrition in older age a multifactorial issue that demands urgent attention [1]. The consequences of malnutrition extend beyond nutrient deficiencies. It is strongly associated with frailty, sarcopenia, impaired immunity, and delayed recovery from illness, leading to increased falls, hospitalizations, and mortality. Early identification and intervention are essential to mitigate these risks and improve quality of life for older adults. Traditionally, malnutrition has been associated with undernutrition and nutrient deficiencies. However, overnutrition—excessive energy intake leading to overweight and obesity—is increasingly prevalent among older adults and significantly affect health outcomes [2].

This Special Issue of Nutrients brings together original research and reviews that advance our understanding of malnutrition in aging populations and propose strategies for prevention and management. Jamroz et al. (Contribution 1) validated the Polish version of the Eating Assessment Tool-10 for early identification of dysphagia. Lee et al. (Contribution 2) explored how social isolation and functional decline influence nutritional status, highlighting the need for integrated care models. Bell et al. (Contribution 3) demonstrated how personalized nutritional interventions can improve recovery post-hospitalization in patients with fragility fractures. Ulambayar et al. (Contribution 4) and Zurashvili et al. (Contribution 5) examined socio-demographic and behavioral risk factors for overnutrition in older Hungarians, identifying modifiable contributors. Two reviews addressed emerging topics: Azzolino et al. (Contribution 6) explored the oral–gut microbiota axis as a mediator of frailty and sarcopenia, while Barbarossa et al. (Contribution 7) highlighted the clinical relevance of non-severe hypophosphatemia in older adults.

Even though significant progress has been made, several gaps remain. Future research should focus on developing digital health tools for real-time nutritional monitoring, integrating artificial intelligence to personalize dietary interventions, and exploring novel biomarkers for early detection of malnutrition and sarcopenia. Longitudinal studies are needed to assess the long-term impact of nutritional strategies on functional independence and healthcare costs. Additionally, research should address cultural and socioeconomic disparities in access to nutrition, ensuring that interventions are equitable and scalable across diverse populations.

Collectively, these contributions underscore the need for a multidisciplinary approach that integrates clinical care, personalized nutrition, social support, and public health initiatives. Promoting adequate nutrition in older adults not only enhances physical and emotional well-being, but also reduces healthcare costs and supports independence.

## Data Availability

No new data were created or analyzed in this study.
